# Supplementary material for: Highly Pathogenic Avian Influenza (H5N1) Landscape Suitability Varies by Wetland Habitats and the Degree of Interface between Wild Waterfowl and Poultry in India
Source: Viruses. 2020 Nov 11;12(11):1290. doi: 10.3390/v12111290 (PMC7697759; doi:10.3390/v12111290)
Supplement: Supplementary file 1 [file viruses-12-01290-s001.pdf]

Table S1. Anatidae species niche comparisons based on boosted regression tree models. Each species listed represents that species' modelled niche with the associated number of observations of the species in the field, model performance (AUC), and individual niche overlap with all other niches.

**Wild Waterfowl**	**Number of field observations**	**AUC (%)**	**Niche overlap (%)**
**Migratory species**			
Anas acuta	19601	89.7	96.4
Anas clypeata	22192	90.4	95.6
Anas crecca	15187	86.4	97.7
Anas penelope	8975	89.8	93.9
Anas platyrhynchos	3128	88.6	90.4
Anas querquedula	20545	93.4	91.5
Anas strepera	11010	88.2	96.3
Anser anser	5173	92.5	93.0
Anser indicus	9754	90.1	96.2
Aythya ferina	7075	89.7	91.5
Aythya fuligula	4413	89.7	91.5
Aythya nyroca	3521	90.3	93.5
Mareca falcata	156	95.7	75.8
Mergus merganser	1143	97.0	67.8
Netta rufina	3979	90.4	91.2
Tadorna ferruginea	16646	87.4	97.2
Tadorna tadorna	566	90.8	88.8
**Composite migratory niche**		88.7	n/a
**Resident species**			
Anas poecilorhyncha	55763	91.0	98.8
Asarcornis scutulata	156	99.7	52.1
Dendrocygna bicolor	2604	98.1	80.0
Dendrocygna javanica	46521	92.3	98.1
Nettapus coromandelianus	14665	92.7	94.6
Sarkidiornis melanotos	9174	90.5	94.4
**Composite resident niche**		90.9	n/a

Table S2. Crude, bivariate regression coefficients and 95% confidence intervals for the associations between highly pathogenic avian influenza virus, subtype H5N1, outbreaks and each landscape feature as derived from an inhomogeneous Poisson model with only the one feature included.

**Landscape feature**	**AIC**	**Coefficient**	**95% confidence interval**	**p-value**
Null model	388.3			
**Climate**				
Mean dry quarter precipitation (10 mm)	350.7	0.04	-0.04 – 0.01	0.30
Mean wet quarter precipitation (10 mm)	346.1	0.004	0.001 – 0.008	0.009
Mean cold quarter temperature (C)	323.1	0.16	0.09 – 0.23	0.00001
Mean warm quarter temperature (C)	348.7	0.04	-0.01 – 0.10	0.12
Mean annual temperature (C)	331.7	0.21	0.07 – 0.35	0.003
**Hydrogeography**				
Distance to freshwater marsh (5 km)	348.0	0.02	-0.11 – 0.16	0.73
Distance to intermittent wetlands (5 km)	343.7	0.30	0.03 – 0.56	0.03
Distance to lakes (5 km)	309.0	-5.25	-7.37 – -3.13	<0.00001
Distance to reservoirs (5 km)	346.5	-0.13	-0.34 – 0.08	0.22
Distance to rivers (5 km)	329.9	-0.89	-1.35 – -0.43	0.0002
Distance to coastal marsh (5 km)	318.1	-0.42	-0.58 – -0.26	<0.00001
Distance to any surface water (5 km)	317.4	-0.30	-0.44 – -0.16	0.00004
**Bird hosts**				
Wild migratory waterfowl niche (%)	313.8	3.50	2.38 – 4.62	<0.00001
Wild resident waterfowl niche (%)	313.5	3.34	2.30 – 4.39	<0.00001
Mergus merganser niche (%)	348.4	1.35	-11.24 – 13.94	0.83
Mareca falcata niche (%)	345.9	43.76	0.58 – 86.95	0.05
Asarcornis scutulata niche (%)	346.4	-150.01	-437.52 – 137.51	0.31
Poultry density (deciles)	340.3	0.14	0.06 – 0.23	0.001

Figure S1. Climate feature distributions.

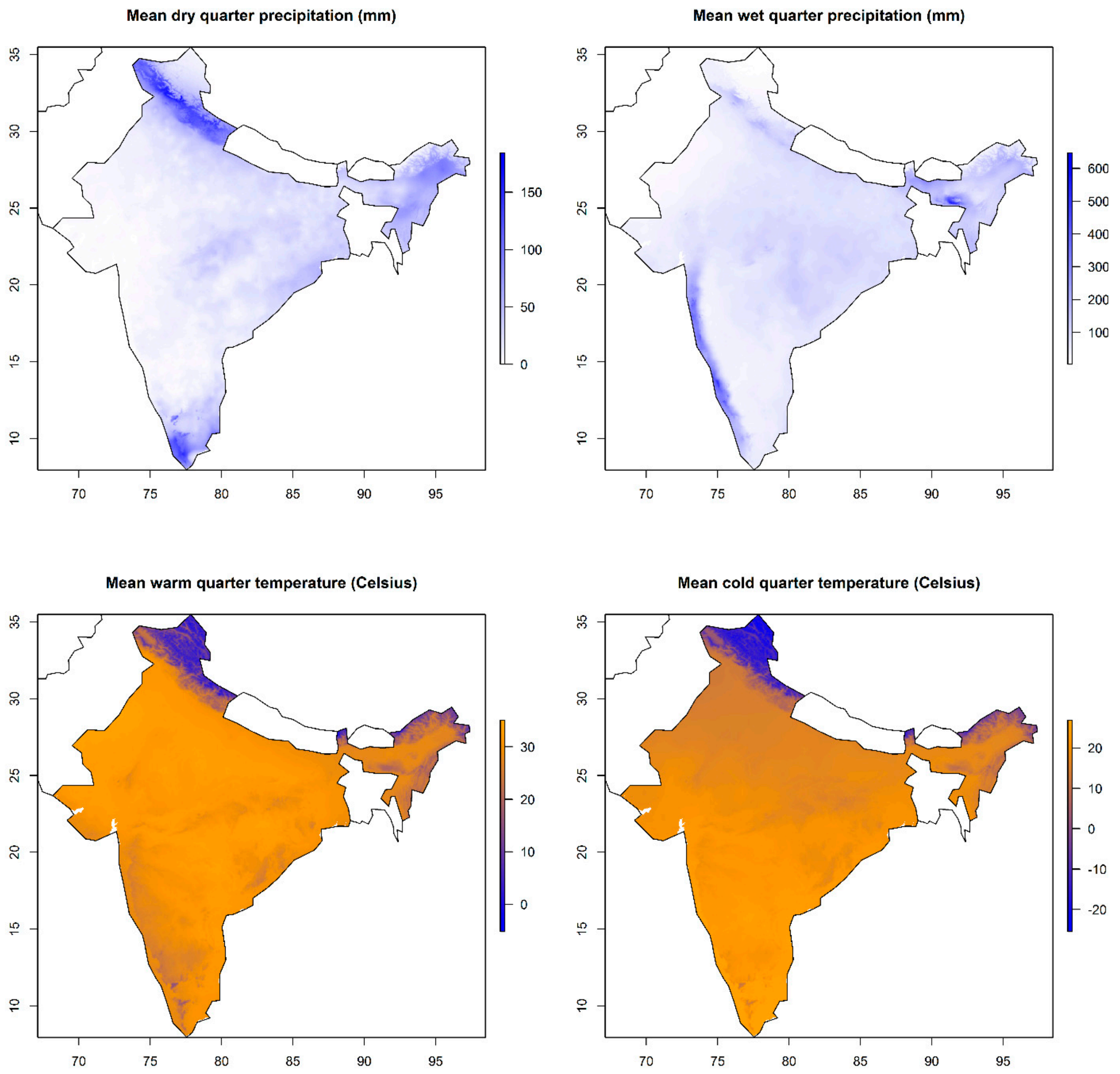

Figure S2. Hydrogeographic feature distributions.

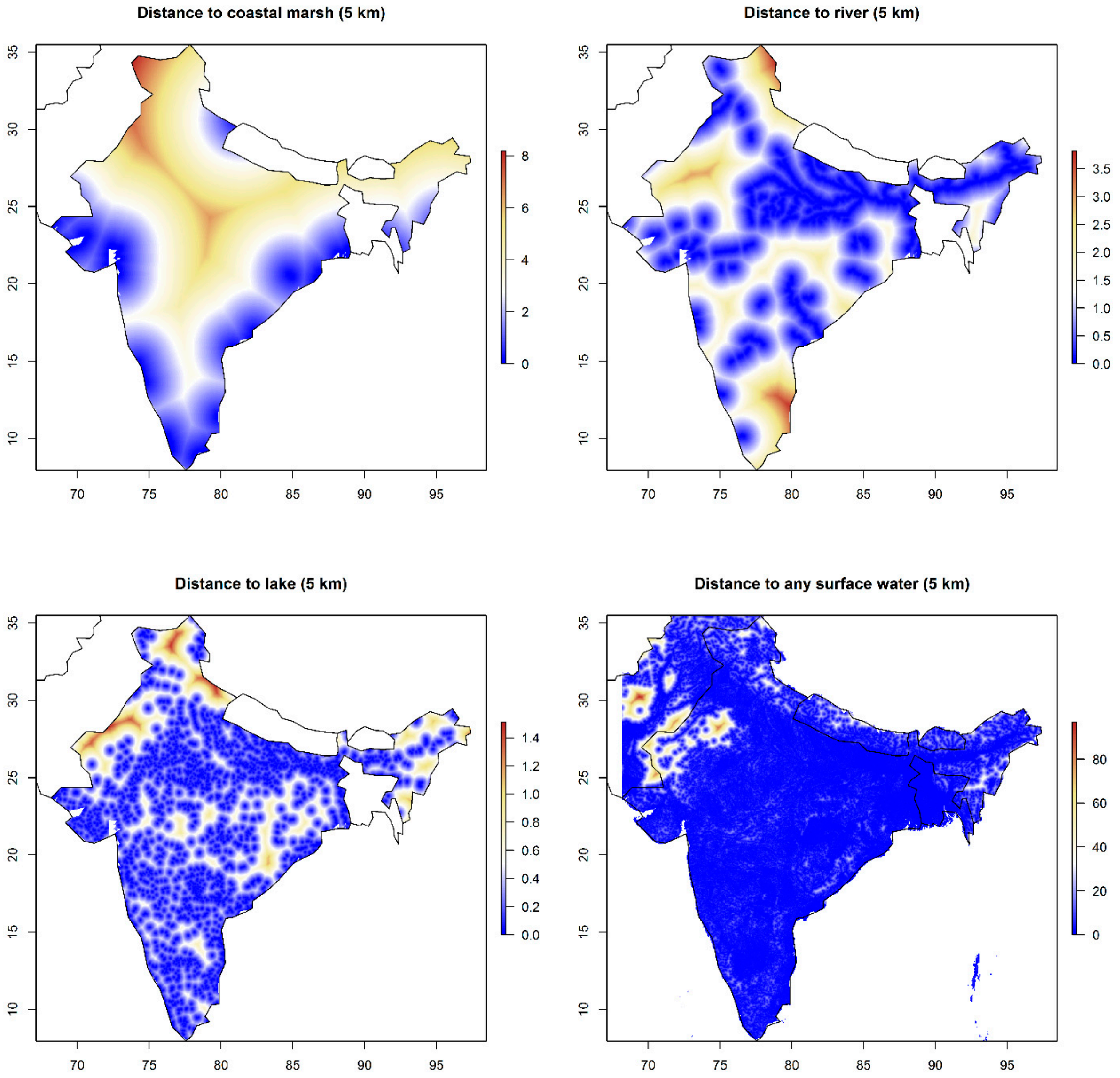

Figure S3. Host feature distributions.

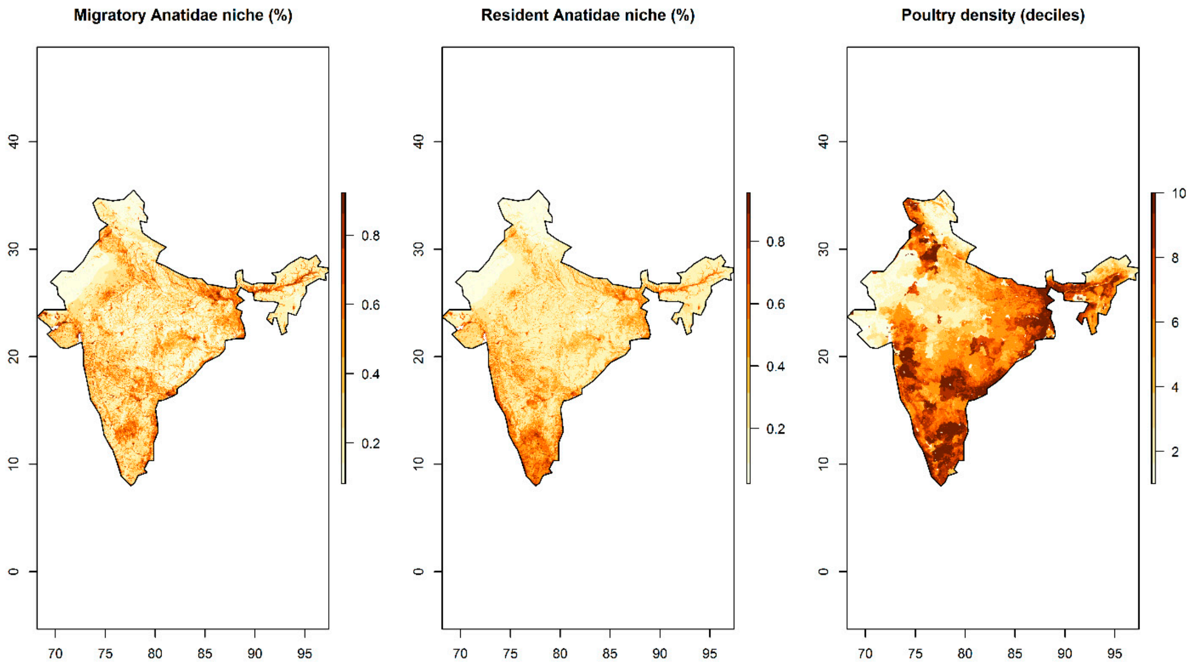

Table S3. Highly pathogenic avian influenza virus, subtype H5N1, inhomogeneous Poisson process model comparisons by Akaike information criterion (AIC) and area under the receiver operating characteristic curve (AUC). All models are presented at fine (2.5 arc-minutes) and coarse (10.0 arc-minutes) scale. Each nested multiple point process model includes those variables that were bivariately associated with H5N1 outbreaks (Table s2).

Pont process models	Scale	AIC	AUC (%)
**Model 1 (Climate only)**: *Mean wet quarter precipitation + Mean cold quarter temperature*			
	2.5 arc minutes	323.4	40.4
	10.0 arc minutes	333.4	39.5
**Model 2 (Surface water only, #1)**: *Coastal marsh proximity + lake proximity + river proximity*			
	2.5 arc minutes	277.6	78.8
	10.0 arc minutes	294.7	81.0
**Model 3 (Surface water only, #2)**: *Proximity to any surface water*			
	2.5 arc minutes	317.4	76.3
	10.0 arc minutes	331.3	79.2
**Model 4 (Reservoirs only, #1)**: *Wild resident waterfowl + poultry density (deciles)*			
	2.5 arc minutes	306.5	77.0
	10.0 arc minutes	322.3	82.0
**Model 5 (Reservoirs only, #2)**: *Wild migratory waterfowl + poultry density (deciles)*			
	2.5 arc minutes	308.0	79.8
	10.0 arc minutes	314.2	84.7
**Model 6 (Full, #1)**: *Mean wet quarter precipitation + Mean cold quarter temperature + Coastal marsh proximity + lake proximity + river proximity + wild resident waterfowl + poultry density (deciles) + resident waterfowl:poultry interaction*			
	2.5 arc minutes	274.6	81.1
	10.0 arc minutes	292.7	86.0
**Model 7 (Full, #2)**: *Mean wet quarter precipitation + Mean cold quarter temperature + Coastal marsh proximity + lake proximity + river proximity + wild migratory waterfowl + poultry density (deciles) + migratory waterfowl:poultry interaction*			
	2.5 arc minutes	275.0	81.3
	10.0 arc minutes	288.0	86.3
**Model 8 (Final, #1)**: *Coastal marsh proximity + lake proximity + river proximity + wild resident waterfowl + poultry density (deciles) + resident waterfowl:poultry interaction*			
	2.5 arc minutes	270.8	82.1
	10.0 arc minutes	287.7	85.9
**Model 9 (Final, #2)**: *Coastal marsh proximity + lake proximity + river proximity + wild migratory waterfowl + poultry density (deciles) + migratory waterfowl:poultry interaction*			
	2.5 arc minutes	271.7	82.9
	10.0 arc minutes	288.7	88.6

Figure S4. Highly pathogenic avian influenza (HPAI) virus, subtype H5N1, landscape suitability based on predicted intensity at 10 arc-minutes (approximately 20 km). The centre panels depict HPAI H5N1 suitability for resident-specific (top) and migratory-specific (bottom) waterfowl models as deciles of the predicted intensities from the best fitting and performing inhomogeneous Poisson point process models (S6 Table 3). The left and right panels depict the lower and upper 95% confidence limits, respectively, for the predicted intensities. Map axes represent degrees of longitude (x-axis) and latitude (y-axis). All maps are displayed only for the purposes of depicting the distribution of disease occurrence and risk, and do not reflect the authors' assertion of territory or borders of any sovereign country including India.

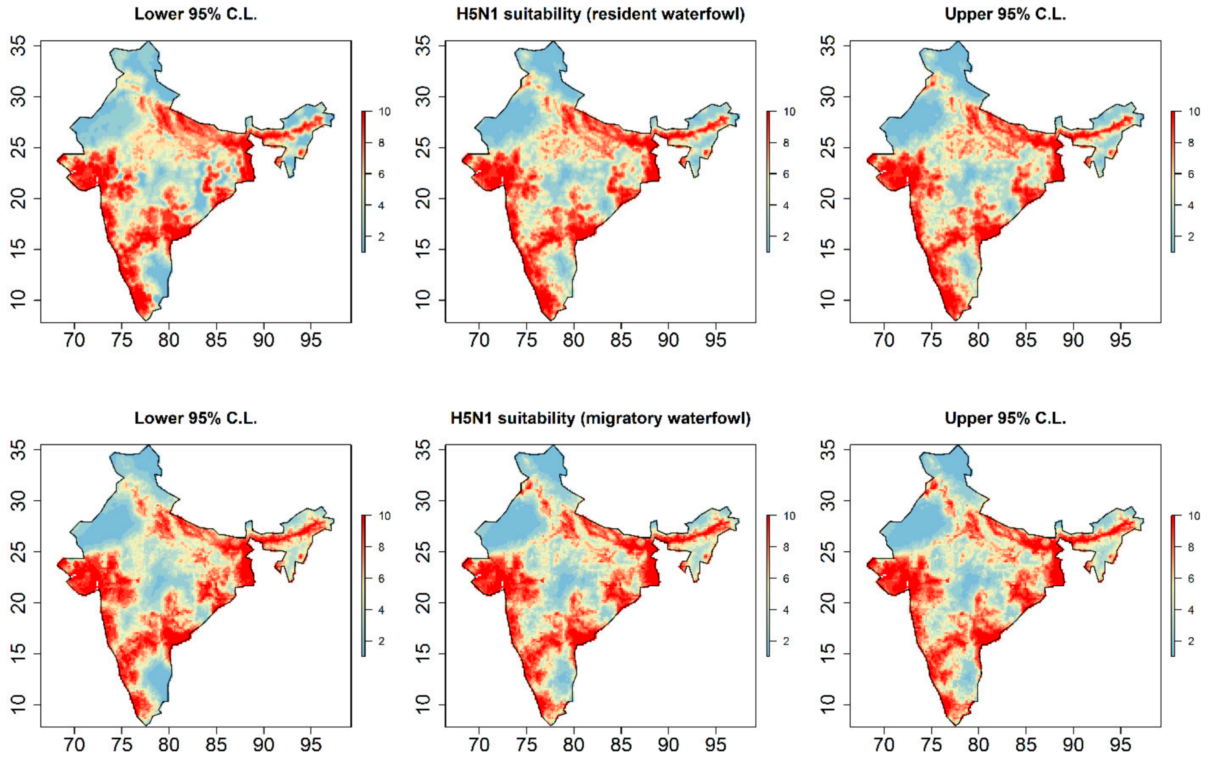

Figure S5. Homogeneous (left panels) and inhomogeneous (right panels) K-functions for the highly pathogenic avian influenza (HPAI) virus, subtype H5N1, outbreak point process at 10 arc minutes. The homogeneous K-function is not an appropriate fit due to the spatial dependency in HPAI H5N1 outbreaks as depicted by the divergent empirical (black line) and theoretical (under spatial randomness; dashed red line with confidence bands in grey) functions. In contrast, the resident (top) and migratory (bottom) waterfowl model-based inhomogeneous K-functions show that the spatial dependency was accounted for by the model covariates (overlapping empirical and theoretical functions). The x-axes, *r*, represent increasing radii of subregions of the window of HPAI H5N1 outbreaks, while the y-axes represents the K-functions.

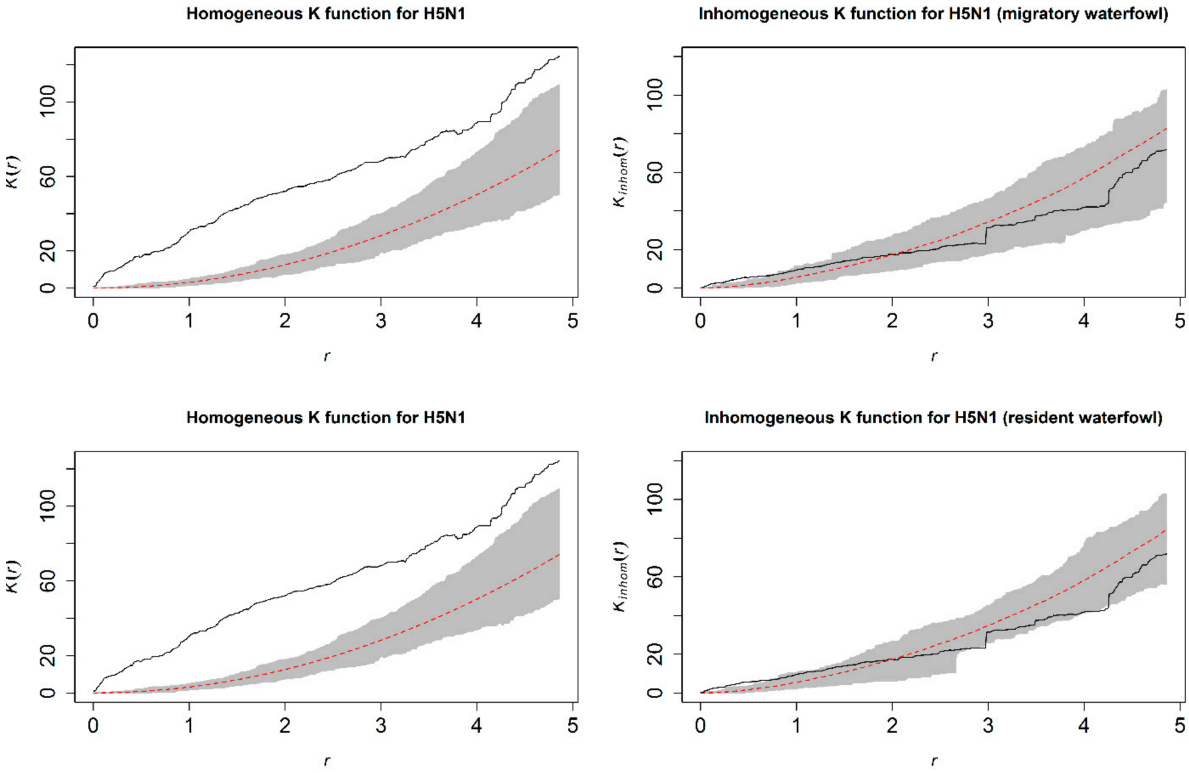

Table S4. Adjusted relative risks and 95% confidence intervals for the associations between highly pathogenic avian influenza virus, subtype H5N1, outbreaks and each landscape feature as derived from the best fitting inhomogeneous Poisson models for resident and migratory waterfowl, respectively. Each landscape feature is adjusted for all others in each of the two models. Models are at a scale of 10 arc minutes (~20 km).

Landscape feature	Relative risk	95% confidence interval	p-value
Model 1: with resident waterfowl			
Resident wild waterfowl composite niche (%)	11.94	2.43 – 58.60	0.001
Distance to coastal marsh (20 km)	0.78	0.66 – 0.92	0.002
Distance to lake (20 km)	0.09	0.01 – 0.76	0.01
Distance to river (20 km)	0.38	0.24 – 0.60	0.00002
Model 2: with migratory waterfowl			
Migratory wild waterfowl composite niche (%)	43.53	9.66 – 196.06	<0.00001
Distance to coastal marsh (5 km)	0.75	0.64 – 0.88	0.0002
Distance to river (5 km)	0.39	0.25 – 0.60	0.00001
